# Supplementary material for: Are carbon emissions trading and green financial instruments synergistic? -Comprehensive quantitative research based on content analysis
Source: PLoS One. 2024 Mar 7;19(3):e0298601. doi: 10.1371/journal.pone.0298601 (PMC10919601; doi:10.1371/journal.pone.0298601)
Supplement: S1 Data — (ZIP) [file pone.0298601.s001.zip › Data/地级市Z_F工作报告.zip!/╜¡╬≈/╞╝╧τ╩╨/└┤╫╘╣1⁄2╓┌║┼í╛┬φ┐╦╩2╛▌═°í┐_2018.docx]

各位代表：

现在，我代表市人民政府向大会报告工作，请予审议，并请各位市政协委员和列席会议的同志提出意见。

一、2017年的工作回顾

2017年是党的十九大胜利召开之年，也是我市新一届政府班子全面履职的第一年。一年来，在省委、省政府和市委的坚强领导下，在市人大、市政协的监督支持下，全市上下认真学习贯彻党的十九大精神，按照“年年有变化、三年大变样、五年新跨越”总体要求，牢牢把握稳中求进工作总基调，以供给侧结构性改革促进经济转型升级，统筹做好贯彻新理念培育新动能、加快城乡发展、改善生态环境、落实民生实事、维护社会稳定等重点工作，全面完成了市十五届人大二次会议确定的目标任务，迈出了决战全面小康的坚实步伐。预计全年实现生产总值1120亿元，同比增长8.8%；规模以上工业增加值同比增长8.9%；财政总收入146亿元，同比增长7.8%;全社会固定资产投资同比增长12.8%；实际利用外资同比增长6.6%；外贸出口同比增长6.5%；社会消费品零售总额378亿元，同比增长12%；城镇居民人均可支配收入33080元，同比增长8%；农村居民人均可支配收入16572元，同比增长8.5%。市十五届人大二次会上确定的20个重大产业项目、20个重大基础设施和城建项目、20件重大民生实事均较好地完成了年度任务。过去一年，政府主要抓了六个方面的工作：

（一）产业转型升级持续发力

工业经济稳中向好。工业经济指标保持向上势头，在全省排位不断前移，预计工业增值税增长30%，工业固定资产投资增长13.5%。传统产业提质增效，重点企业态势向好。新兴产业加速崛起，预计战略性新兴产业实现增加值同比增长10.5%，较规上工业增速高1.6个百分点；高新技术产业实现增加值同比增长11%，较规上工业增速高2.1个百分点，占规上工业的41%。

农业发展活力增强。生态高效特色农业加快发展，高标准农田建设扎实推进，预计全市实现农业增加值57亿元，增长4%。新型农业经营主体比上年增加453个，达到5535个，其中市级以上龙头企业433个，增加42个；“三品一标”认证企业95家、产品114个；现代种业、草食畜禽、设施农业等特色产业不断壮大，南繁制种面积再创历史新高，占全国面积85%以上。休闲农业发展势头良好，休闲农业点达到625个，比上年增加78个，芦溪仙凤三宝园成为我市首个省5A级乡村旅游点，安源五陂红旗分场被农业部评为中国美丽休闲乡村。莲花县被农业部批准为国家农产品质量安全创建试点县。

现代服务业快速发展。预计第三产业完成增加值457亿元，增长10%。全市接待游客人次同比增长20%，实现旅游综合收入同比增长27%。武功山景区以创建国家5A级旅游景区为目标，游客服务中心、石鼓寺、金顶核心景区等重点项目全面建成，硬件设施和服务水平全面提升。安源景区被评为十大国家工业遗产旅游基地，安源纪念馆跻身国家一级博物馆并入选全国红色旅游经典景区名录。杨岐山景区获评国家级风景名胜区。商贸物流、文化体育、健康养老产业加快发展，电商交易额预计同比增长30.6%，江西快乐体育产业园、湘东包装创意产业等新业态蓬勃发展，都市农庄生态园开发有限公司被评为全省唯一一家“智慧健康养老应用示范企业”。金融业发展迅速，直接融资工作取得突破，开发区在全省首发20亿元绿色债券。

此外，在过去的一年里，全市不断优化产业结构，产业转型升级受到国务院通报表彰并呈现出良好态势，主要表现在：

创新能力不断提升。大力实施创新驱动发展战略。与18家国内高等院校、科研院所签订战略合作协议，共建发展研究院（技术转移中心）达到7家，科技成果转化110余项。省级工程技术研究中心达到22个，比上年增加5个；国家高新技术产业化基地达到5个；省级科技协同创新平台达到9个。国家高新技术企业达到76家，同比增长52%。全市发明专利申请量370件，同比增长102%。中材电瓷特高压支柱瓷绝缘子生产技术改造工程荣获全国建材行业技术革新一等奖。

项目建设力度空前。全市确定重点项目120个，总投资1028亿元。列入省重点建设项目17个，项目数列全省第三。重大产业项目推进有力，广利通科技、岳峰环保、中材风电叶片（二期）、尚立动力（一期）、麓林湖养生公馆（一期）等一批重大项目建成投运，亿丰伟业、欧派斯润滑油、天虹创业创新基地等一批重大项目开工建设，熙谷小镇、佳禾文化书城广场、胜龙牛业等一批重大项目全面推进。

产业集群逐渐形成。预计工业产业集群完成工业增加值同比增长10.5%。市级产业发展引导基金成功创立，工业陶瓷、电瓷、花炮等传统产业集群加速改造提升，电子信息、食品医药、节能环保等新兴产业集群逐渐壮大，中材电瓷电气、甘源食品、格丰科技等产业龙头加快发展。经开区获批国家循环化改造重点支持园区。湘东区工业陶瓷产业被工信部列为全国产业集群区域品牌建设试点。芦溪县获国家电瓷知名品牌创建示范区，上栗县列入国家第二批增量配电业务改革试点县。

新经济茁壮成长。新经济项目推进扎实有力，“双新”项目达到188个。新制造经济蓬勃发展，绿色经济初具规模，智慧经济开始萌芽，培育了企创、飞天等一批电商创业创新基地和一家国家级众创空间，网是科技、丝路科技、泛海统联等智慧企业茁壮成长，经开区科技金融等新经济业态稳健发展。

（二）改革开放迈出新步伐

重点领域改革取得实效。积极推进供给侧结构性改革，煤炭产业化解产能30万吨，关闭煤矿6家，超额完成年度任务。稳妥推进财税体制改革，预决算公开更加规范，财政资金盘活力度不断加大。推进基层市场监管规范化建设，市场监督体制改革得到深化。全省“多规合一”试点改革推进有力，编制完成《萍乡市城乡总体规划（多规合一）（2015-2030）》。医药卫生体制改革不断深化，健康扶贫工程卓有成效，药品加成全面取消，分级诊疗、家庭医生签约服务等改革扎实推进，芦溪县级公立医院改革受到国务院通报表扬。农村土地改革有序推进，农村土地承包经营权确权登记颁证工作走在全省前列，土地流转率达到50.9%，创历史新高。

招商引资取得良好成效。预计引进2000万元以上省外资金项目215个，实际进资472亿元，同比增长10%。中国建材产业园、中国先进工业陶瓷产业园分别落户芦溪县、湘东区，世界500强恒大集团、碧桂园落户经开区。成功举办首届世界萍商大会暨“一带一路”产业合作发展论坛，签约项目65个，签约资金达296亿元。

区域开放合作不断推进。赣湘开放合作得到深化，赣湘两省已形成推动开放合作试验区上升为国家战略的共识，湘东、上栗园区建设提速，项目引进和公共平台建设取得实效。成功承办省政府第三届赣西经济转型加快发展区域合作会议，旅游、生态文明等领域合作有效推进。口岸平台建设取得突破，引进广州港务集团在湘东设立了内陆“无水港”。

（三）统筹城乡发展成效显著

海绵城市建设成效明显。海绵城市完工项目121个，完工面积达26.511平方公里，城市水生态系统得到重塑，玉湖、萍水湖等重点项目开始发挥作用，城区主次干道和小街小巷海绵化改造全面推行，“逢雨必涝、内陆看海”顽疾得到改善，正逐步实现“小雨不积水、大雨不内涝、水体不黑臭、热岛有缓解”目标。在2016年度全国海绵城市建设绩效考评中再次名列第一。海绵城市建设得到了全市人民的点赞，萍乡经验和模式获得人民日报、中央电视台、新华社等媒体聚焦报道，正从全国试点走向全国示范。

城乡基础设施日益完善。交通更加高效便利，萍莲高速项目实现开工，成功争取厦渝铁路长赣段项目经过萍乡并设站，中环北路、杨宣公路等“硬骨头”项目取得突破性进展，中环西路即将实现通车。国省道路升级改造和县、乡、村公路建设扎实推进，完成国省道路升级改造里程71.3公里，农村公路升级改造150公里。城区背街小巷“白改黑”全覆盖工程取得显著成效，改造背街小巷466条，得到上级领导和萍城百姓的称赞。城市承载力得到增强，城市垃圾焚烧发电项目、五陂下水厂提质改造项目建成运行，公交站场、地下综合管廊进一步完善。城市品位正在提升，南正街综合改造、城市大厦、鳌洲公园等一批城市地标项目有序推进，五陂海绵小镇、下埠工陶小镇等省、市特色小镇建设积极推进。

城市管理水平不断提升。以争创全国文明城市为抓手，大力推进城乡环境综合整治，市容乡貌实现了大变样，文明创建深入人心，文明程度明显提升。全力推进“两违”特别是高铁沿线乱搭乱建专项整治，实现“零新增、零容忍、控增量、去存量”目标。超载撒落、道路和工地扬尘整治全面从严并取得明显成效。深入推进城市管理智慧化、环卫作业市场化、城市执法综合化、队伍建设正规化，城市管理更加高效有序。

新农村建设步伐加快。围绕建设整洁美丽和谐宜居乡村目标，以“六统·四联创”活动为抓手，全域推进秀美乡村建设，在全市835个自然村开展新农村建设工作，惠及农户11.6万户、34.8万余人，打造了319和320国道沿线美丽示范风景线、芦万武旅游公路示范带、下埠镇潘塘村、高坑镇楠木村、六市乡美丽乡村、芦溪镇东阳村、赤山镇幕冲村等一批秀美乡村典型示范。

（四）社会民生事业有力推进

脱贫攻坚全力推进。坚持将脱贫攻坚作为重大政治任务和头号民生工程推进，不断强化合力攻坚，贫困识别和退出更加精准，帮扶措施更加扎实，资金拨付更加高效，基层基础更加牢固，全年实现23000名以上贫困人口脱贫、62个贫困村退出，圆满完成了脱贫攻坚年度目标任务，贫困地区面貌和群众生产生活条件发生了可喜变化，贫困群众的生活质量明显提升。

民生实事成效显著。统筹安排财政资金50.4亿元，集中办好20件民生实事，特别是普通高中阶段免学费、65岁以上老年人免费乘坐公交车、城乡居民免费参加大病保险等“八个全覆盖”如期兑现。就业形势稳定向好，预计全市城镇新增就业3.3万人，困难群体就业3100人，新增转移农村劳动力2.3万人，均超额完成年度目标任务。创业环境不断改善，安源区、湘东区、上栗县获批“全国返乡创业试点地区”。棚户区改造项目开工率100%，居全省前列。社会保障体系更加完善，被征地农民养老保险工作在全省率先取得突破，近70%的被征地农民实现参保。兜底性社会救助保障更加牢固，城乡低保、农村五保、城镇“三无”人员、孤儿等困难群众保障标准全面提升。

社会事业协调发展。教育事业全面加强，芦溪县被列为全国学前教育改革实验区，萍乡在全省率先通过国家县域义务教育基本均衡发展评估验收，高考本科升学率保持全省先进，大力支持了萍乡学院发展，萍乡卫生职业学院列入省“十三五”高校设置规划。医疗卫生服务体系不断完善，市第二人民医院、萍矿总医院评为三级甲等综合医院，全市三甲医院达到5家，居全省前列。人口计划和目标任务全面完成，全面两孩政策稳妥实施。食品、药品、烟草等市场监管更加严格、秩序更加规范。法治建设全面加强，公共法律服务体系建设扎实推进。文化事业繁荣发展，电影《老阿姨》获得全国“五个一工程”奖。体育事业快速发展，全民健身扎实推进，成功举办市第十三届运动会，萍乡籍运动员在天津全运会上获二银一铜的佳绩。

安全稳定维护有力。安全生产形势持续平稳，安全生产事故和伤亡人数继续下降,非煤矿山和危险化学品两大高危行业实现“双零”目标。荷尧“11·27”事件应急处置及时有力，得到社会各界高度称赞。国防后备力量建设不断加强，军民融合发展取得实效。消防安全工作扎实有效，全年无亡人火灾事故发生。信访工作成效明显，位列全省前茅。社会治安大局持续平稳可控，全年发案率下降，打击处理率上升，反盗抢专项行动、文明交通行动、禁毒社会化等成效显著，确保了十九大期间社会安全稳定，公众安全感和对公安工作满意度居全省前列。

（五）生态文明建设积极有效

打响了净空净水净土攻坚战。扎实推进“蓝天碧水·2017行动计划”，突出抓好环境污染治理。强化工业污染源监管，严格城市扬尘污染管控，积极应对重污染天气，实现PM10、PM2.5“双下降”和空气优良率“提升”的目标。深入实施“河长制”，加强城市饮用水水源环境保护工作，饮用水水源地水质达标率为100%；持续开展清河行动，推进新华河路行、萍水河南门桥等四个断面消灭劣V类水整治专项行动，局部地区水质状况逐步改善。切实加强土壤、危险废物等各类污染防控，全年无重大环境污染事故。

大力推进了生态建设。全国水生态文明城市试点建设有力推进，地表水功能区水质达标率达到89.3%，连续6年达到省政府下达的控制指标要求。创建国家森林城市工作有序推进，确定了香樟树为“市树”、杜鹃花为“市花”，森林覆盖率达到67.24%，位居全省第三。森林药材、森林旅游等林下经济发展迅速，白竺青草湖景区获评我省首批森林体验基地。莲花县成为国家生态文明试验区（江西）生态扶贫试验区之一、全省生态文明十大领跑县。

（六）政府自身建设不断加强

“放管服”改革持续推进。深入实施“三单两制一网一评价”，大力精简行政权力事项31项。深化商事制度改革，“多证合一”全面实行。积极实施“省130条”和“市121条”等惠企政策，降低税费成本、用能成本，累计为企业减负约24亿元，在政府主导、企业支持下，萍乡天然气工业用气价格实行阶梯价格，最低降至2.6元/?。深入推进政务服务事项集中办理，完善“互联网+政务服务”，做到让数据多跑路、群众少跑腿。

依法行政深入推进。自觉接受人大监督、政协民主监督，办理人大代表议案、建议68件，政协提案103件，办结率100%。完善依法行政制度体系，落实重大决策合法性审查制度。坚持规范公正文明执法，实现行政检查“双随机”工作制度化、规范化、信息化，积极落实行政执法与刑事司法衔接信息共享平台建设。

廉洁从政得到加强。持之以恒纠正“四风”，深入开展基层“微腐败”专项治理。持续保持惩治腐败高压态势，重点查处了一批民生领域特别是扶贫领域违纪违规行为，严肃问责了一批履职不力的部门和人员。开展政府系统全面从严治党“两个责任”专题约谈，加强对政府部门工作人员纪律教育和廉政教育。加大了对领导干部的离任审计力度，首次开展了领导干部自然资源资产离任审计，推进风清气正的政治生态建设。

与此同时，一年来，统计、国家安全、决策咨询、国资监管、民族宗教、外事侨务、档案管理、涉台事务、地方(史)志、接待、老龄、国防动员、民兵预备役、防震减灾、驻外机构、住房公积金等各项工作取得了新进步；气象、无线电管理、石油、盐务、邮政、人行、银监、保险等中央、省驻萍单位为全市经济社会发展作出了新的贡献。

各位代表，过去一年的成绩，是省委、省政府和市委正确领导的结果，是市人大、市政协监督和支持的结果，是全市人民辛勤与智慧的结果。在此，我代表市人民政府，向全市人民，向各民主党派、工商联、无党派人士、人民团体，向驻萍部队、武警官兵和公安民警，向国内外所有关心支持萍乡发展的各界朋友，表示衷心的感谢和崇高的敬意！

回顾过去一年，我们也清醒地看到，我市经济社会发展中还存在不少困难和问题。主要表现在：经济总量偏小，经济质量不高，产业结构不优，新兴产业竞争力还不强，经济转型升级任务仍然艰巨；城市建设还存在欠账，城市管理水平有待提升，城乡私搭乱建的现象依然存在，城乡环境综合治理成果有待巩固；民生事业还有短板，脱贫攻坚工作任务繁重，与全面小康社会的目标仍有差距；环境保护、安全生产、社会稳定等方面的形势仍然严峻；政府服务水平与人民群众期望还有差距，发展环境有待进一步优化，等等。这些困难和问题亟待我们在新时代的发展中努力解决。

二、2018年的主要任务

2018年是全面贯彻党的十九大精神的开局之年，是改革开放40周年，也是我市决胜全面小康和实现“三年大变样”承上启下的关键一年，做好政府各项工作至关重要。今年政府工作的总体要求是：全面贯彻落实党的十九大精神，以习近平新时代中国特色社会主义思想为指导，按照中央和省委、省政府的重大决策部署，在市委的正确领导下，围绕“年年有变化、三年大变样、五年新跨越”，坚持稳中求进工作总基调，着力贯彻新发展理念，着力推动工业经济、现代农业和现代服务业加快发展，着力统筹城乡发展、脱贫攻坚、生态文明、民生事业、防范风险、安全稳定各项工作，为决胜全面建成小康社会、谱写富裕美丽幸福现代化江西的萍乡新篇章而努力奋斗。

今年全市经济社会发展的主要预期目标是：生产总值增长8.5%左右，财政总收入增长7%左右，全社会固定资产投资增长11%以上，规模以上工业增加值增长8.5%左右，实际利用外资增长6%左右，外贸出口增长2%左右，社会消费品零售总额增长12%左右，城镇居民人均可支配收入增长8%左右，农村居民人均可支配收入增长8.5%左右，居民消费价格总水平涨幅控制在3%以内，万元工业增加值能耗下降2%，城镇登记失业率控制在3.85%以内。

为实现上述目标，今年政府将主要抓好八个方面的工作：

（一）培育壮大产业集群，推动新经济大发展

以培育产业集群为重点做强做大实体经济。始终把培育壮大产业集群作为工业转型升级的头号工程。精准改造传统产业集群：改造提升电瓷、工业陶瓷、建材等产业；重组改造花炮、冶金产业；逐步退出煤炭产业，加快淘汰落后产能，为新动能发展腾出空间。突出培育新兴产业集群，要持之以恒培育壮大一批产业关联、产能倍增、产品高端的主导产业集群，推进产业供给质量提升和品牌建设，推动一批战略性新兴产业迅速崛起。各县区、经开区要突出自己首位产业，既错位发展，又联动配套，形成全市范围的新一代电子信息、先进装备制造、节能环保、食品医药和海绵产业等五大新兴产业集群，迈出萍乡产业升级的坚实步伐。

以培育龙头企业增强产业带动力。始终将育龙头作为培育产业集群的重中之重。大力扶持以中材风电装备、格丰科技、甘源食品等新兴产业龙头企业，改造提升萍安钢等传统龙头企业，做到“一企一策”精准扶持，激励企业技改扩能、挖潜增效。

以狠抓重大项目建设增强产业竞争力。抓龙头、育集群必须有一大批重大项目为支撑。各县区及其产业园均要按照“引进落地一批、开工建设一批、竣工投产一批、达产入规一批”的要求，全力推进中材高新、优选时代包装、伊博智能、锐达电子、丝路科技等一批重大产业项目建设（见附件2）。密切跟踪国家、省有关项目扶持政策，为萍乡项目建设争取更多政策资金支持。严格落实项目建设“五个一”要求，不断增强经济发展动力。

以做优工业平台增强产业支撑力。全市各类园区是经济建设的主战场，是全市产业升级的主平台，我们要科学谋划全市工业园区布局，启动新的市级工业平台建设，确定平台运作模式，推进基础设施建设，力争年内建成雏形。加快赣湘开放合作试验区湘东、上栗园区建设，使之成为对接长株潭和实现湘东、上栗乃至全市产业升级的新平台。高标准规划高铁经济示范区，着手打造沪昆高铁、厦渝高铁沿线产业经济带。优化县区工业平台，清理闲置低效用地，加快“腾笼换鸟”，推进土地集约节约利用。推动智慧园区、绿色园区建设，完善园区服务体系，优化企业发展环境。使全市各类园区成为先进制造业、高新技术产业和生产性服务业集聚发展的平台，成为实现创新驱动和发展新经济培育新动能的重要载体。

（二）深入推进改革开放和科技创新，增强经济发展动力和活力

深化重点领域改革。推进商事制度改革，加大“多证合一”改革力度，助力“大众创业、万众创新”。深化医药卫生体制改革，推进医疗、医保、医药三医联动，建立优质高效的医疗卫生服务体系。全面深化城管综合体制改革，推进城市管理智慧化、环卫作业市场化、城市执法综合化、队伍建设正规化。深化林权配套制度改革，强化森林保险和林权抵押，推行林地经营权流转证制度。深化国土资源管理改革，启动自然资源统一确权登记。推进农村集体产权制度改革，完成房地一体的集体建设用地和宅基地使用权的确权登记发证工作。深化国资国企改革，做大市本级国有资产总量。积极稳妥推进事业单位公务用车制度改革。

推进开放型经济发展。紧紧抓住沿海地区产业升级和先进制造业转移难得窗口期，围绕我市重点产业集群，大力实施招商引资重大项目攻坚行动，重点瞄准央企、国企、500强企业、行业龙头企业、上市公司等目标，有针对性制定产业招商政策。巩固和利用萍商大会招商成果，大力促进萍商回归、人才回乡。优化招商引资的奖励政策，加大承诺兑现力度，帮助企业切实解决用地、用工、物流等问题，调动各级政府、社会各界参与招商引资、服务项目建设的积极性，力争在引进投资50亿元、100亿元以上的重大产业项目上有所突破。深入对接“一带一路”、长江经济带、长江中游城市群等国家战略，全力打造口岸平台，推进广州港萍乡内陆“无水港”建设，推动海关在萍乡设立服务窗口，拓展完善上栗公路口岸作业区功能，促进上栗国检监管区扩能增效，力争开通萍乡始发的中欧班列。

深入实施创新驱动战略。聚焦全市重点产业集群，围绕产业链部署创新链，深化产学研合作，建设一批企业技术中心、工程技术研究中心和重点实验室，力争实现技术平台与产业发展“一对一”服务，力争全市新增高新技术企业15家以上，新增省级以上科技创新平台和载体3个以上，万人发明专利拥有量、R&D经费占GDP比重有较大提升，科技对经济增长贡献率达到58%。

（三）实施乡村振兴战略，推动农业农村加快发展

加强农业生产和基础设施建设。将高标准农田建设作为全市农业工作的重要抓手，带动农田水利设施建设、农村土地有序流转和农业产业结构调整。完成好2017至2018年度4.1万亩的建设任务，着力建设一批千亩示范片。坚持路网、水网、电网、田网一体化布局,高起点规划、高水平推进2018至2019年度高标准农田建设工作，确保6.7万亩建设任务分解到位、规划到位、落实到位。启动碧湖潭水库建设，全面建成寒山、功德山、大沙江水库，继续推进31座灾后病险水库除险加固。加大农业高效节水灌溉示范县项目建设，力争全年完成5000亩的高效节水灌溉面积。

大力发展现代农业。聚焦生态高效特色农业，构建“优质水稻+现代种业、草食畜禽、高效蔬菜”的“1+3”特色产业结构。积极推进优质粮食工程，落实粮食安全省长责任制，确保粮食总产43万吨以上。推进稻鱼、稻虾综合种养模式，提升农业综合效益。着力培育新型经营主体，发展休闲农业，认证一批“三品一标”农业企业和产品，提升农业产业化水平。按照“一区多园”建设要求，在5个省级以上现代农业示范区内打造30个市级以上现代农业示范园，培育发展田园综合体，打造武功山现代农业科技产业园等一批典型园区。

扎实推进新农村建设。大力推进农村危房、公路、厕所、圈舍、厨房、水井等六大改造工程，推动城乡基础设施和公共服务向农村延伸覆盖。全面实施村庄规划，严格管控农村建房，让农村的房子看起来更美观、住起来更实用。推进农村环境卫生整治，重点抓好农村生活垃圾和污水治理，整治农业面源污染。加强农村基层基础工作，培养造就一支懂农业、爱农村、爱农民的“三农”工作队伍，从严从实抓好村级“两委”换届工作。加强农村文化遗存保护，创新乡贤文化，发挥村规民约的教化、约束作用。

（四）大力发展现代服务业，增强第三产业的经济拉动力

大力发展旅游业。按照“3+1”的旅游发展思路，突出建设武功山、安源、杨岐山三大景区和发展全域旅游。制定全域旅游发展规划，以萍乡历史上著名的“成功之路”、“胜利之路”为主线，串连萍乡各景区景点，打造800里山水长廊的全域旅游框架。全力推进武功山景区“三环、三区、三中心”建设，确保成功创建国家5A级旅游景区。启动安源景区规划建设和创建国家5A级旅游景区的准备工作，深入推进汉冶萍世界工业遗产申报工作。精心打造武功山户外小镇、下埠工陶小镇、上栗花炮小镇、宣风凤栖小镇、大丰豆腐文化小镇、沿背红色培训小镇等一批全域旅游示范点。确保全市实现旅游接待人次和综合收入分别增长20%和25%以上。

稳定发展金融业。打好防范金融风险攻坚战，坚决做好防范和处置非法集资工作，确保不发生区域性金融风险。将服务实体经济与防范化解金融风险相结合，增强金融对实体经济的支撑力。加快引进金融机构步伐，积极稳妥推进科技金融、大数据金融等新业态，力争把萍乡打造为赣湘边界的科技金融创新试验区。积极利用国际金融机构和国家开发性、政策性金融机构的优惠政策，深化全市中小企业信用体系试验区建设，努力提升萍乡市金融综合服务平台的品牌价值。加快建设金融商务区，尽快形成金融产业集聚效应。积极推进企业上市挂牌，重点支持宝海微元、甘源食品首发上市，争取实现我市本土企业上市“零”突破。努力扩大信贷规模，优化信贷结构，满足企业多样化的金融需求。大力发展现代保险业。

全力扶持发展新业态。做强做大现代商贸物流业。引进一批大型物流企业，着力构筑1至2个智慧物流服务平台，科学论证并积极打造赣西物流中心。加快建成天虹广场、梦想天街、佳禾文化广场等大型商业文化综合体，推进中国供销农产品批发市场等一批大型综合批发市场建设，支持高端旅店业发展。抓好电商平台建设，培育电商消费热点。积极推进中医药养生、现代养老服务等健康产业发展。壮大文化产业，坚持“引进来”，推动文化企业落户萍乡，充分挖掘文化消费市场；坚持“走出去”，瞄准花炮、动漫、包装创意等文化主导产业，加快建设上栗花炮文化博览园、湘东包装创意产业园等文化产业园区，形成具有全国影响力的特色文化产业集群。

（五）打造江南特色海绵城市，提升城市规划建设管理水平

全力建成海绵示范城市。加快推进海绵项目建设，保质保量如期完工，力争在国家三年终期绩效考核中排名前列。加强竣工海绵项目的管理和运营维护，确保海绵项目持续发挥作用，把海绵城市打造成群众满意的民心工程。全面贯彻海绵发展理念，深化海绵城市建设全域试点，将海绵城市理念和技术标准贯穿于城市建设，围绕海绵特色小镇打造海绵产城融合样板，推进萍乡城市转型升级。完善海绵产业发展规划，开展海绵技术研究，大力发展海绵产业和海绵经济，设立海绵产业发展基金，组建萍乡市海绵产业集团，使之成为融海绵城市标准制定、规划设计、工程建设、运营管理、产品研发和生产制造为一体的全海绵产业链的龙头企业，服务于全省乃至全国的海绵城市建设，带动萍乡建材产业转型升级。大力推动萍乡海绵城市建设从全国试点走向全国示范。

实施一批城镇化重大项目。推动《萍乡城乡总体规划（多规合一）（2015-2030）》尽早获得省政府批复并严格执行。全力抓好20个基础设施和城市地标重大项目（见附件3），开工建设玉湖学校、市图书馆、奥体中心等一批重大项目，全力推进萍莲高速、中环路、南正街改造等一批重大项目，竣工投运杨宣公路、翠湖公园、鳌洲公园、地下综合管廊等一批重大项目，实现萍乡城市品质大提升、城市面貌大变样。扮靓一批城市主街道、主路口、主建筑、主景点、主河道，重点打造历史街、文化街、品牌街、步行街等特色街巷。全力打造一批产业发展有特色、功能叠加能聚集、制度供给显活力的特色小镇，确保我市第一批特色小镇建设“一年见雏形、两年成规模、三年显成效”。侯卫东官场笔记

深化城乡环境综合治理。强化规划的权威性，大力开展城市建成区违法用地和违法建设专项治理，完善城乡建房审批流程，保持打击“两违”的高压态势，推动城乡土地集约利用，抓好铁路边、公路边、河湖边、城际边、街巷边等重点区域环境综合整治。打好城市“双修”攻坚战，积极改造一批城中村、成片棚户区和老旧小区，高标准建设一批公共停车场、绿地小游园和健身小广场，不断改善萍乡宜居环境。坚持创建全国文明城市目标不动摇，推进移风易俗，促进乡风文明，增强城乡环境整治内生动力。

（六）推进生态文明建设，努力打造美丽萍乡

加快推进绿色发展。以创建国家产业转型升级示范区为契机，建立健全绿色生产和消费的政策导向，着力降低陶瓷、钢铁、水泥等传统行业能耗和排放。引导培育绿色金融，加大对节能环保、新能源等绿色产业支持。大力发展光伏、水电等可再生能源，构建清洁低碳、安全高效的能源体系。积极发展绿色建筑和装配式建筑，为低碳城市发展助力。推进资源全面节约和循环利用，在全市倡导简约适度、绿色低碳的生活方式，开展创建节约型机关、绿色学校、绿色社区和绿色出行等行动。

积极建设生态文明试验区。构建山水林田湖草城系统保护与综合治理、环境保护与监管、绿色产业发展等各项制度体系。健全责任追究机制、部门协调联动机制，从源头上预防污染和生态破坏。做好水生态文明城市建设试点验收准备工作，努力建成全国水生态文明城市。全力创建国家森林城市，积极对接和建设罗霄山国家森林步道，加快碧湖潭国家森林公园建设，深入开展“三边四沿”植树造林、中心城区公园绿化和裸地植草植树等工作，推进城乡森林绿化“四度”提升，推进森林博物馆、森林科普基地等项目建设，确保今年验收成功。

着力打好污染防治攻坚战。全面实施“蓝天碧水净土·2018行动计划”，继续开展环境污染集中整治活动，确保大气、水、土壤环境整治取得明显成效。推进挥发性有机物、餐饮油烟、机动车尾气、工业废气、工地与道路扬尘、垃圾与秸秆焚烧等污染防治工作，确保我市中心城区空气优良天数比例提高到80%以上，PM10、PM2.5年均值实现“双下降”。深化落实河长制，强化清河行动，加快城镇和工业园区污水处理设施及配套管网建设，开展河库源环境综合整治，确保所有断面全面消灭劣V类水、所有河流水库水质不发生恶化、所有河段和水库达到功能性要求。全面开展土壤污染调查评估工作，编制土壤污染防治工作方案，着力抓好土壤污染治理项目特别是省级农用地土壤污染治理示范项目的实施，确保土壤环境安全。加快完善重点企业污染源监控平台、河长制综合信息平台等项目，逐步实现环境基础数据和质量监测信息化。抓好中央、省环保督察反馈问题整改，组织开展市级第一轮环境保护督察，全面开展领导干部自然资源资产离任审计。

（七）积极实施民生工程，促进社会和谐稳定

坚决打好精准脱贫攻坚战。坚持精准扶贫、精准脱贫，强化党政“一把手”负总责的责任制，深入推进大扶贫格局。注重扶贫与扶志、扶智相结合，加快推进产业扶贫全覆盖、健康扶贫再提升、搬迁扶贫再精准、教育扶贫再对接、贫困村庄整治再推进等重点扶贫工程，举全市之力确保莲花县“脱贫摘帽”，确保完成全市30个贫困村退出、10190名建档立卡贫困人口脱贫的工作目标。

认真办好民生实事。坚持以人民为中心，着力实施以“八大工程”为重点的20件民生实事（见附件4），做到民生实事一件接着一件办、一年接着一年干。推动实现更高质量和更充分就业，重点抓好“去产能”企业职工安置，力争全市城镇新增就业3.3万人，新增转移农村劳动力2.2万人。完善保障性住房的后续管理，全力推进棚户区改造，积极稳妥培育房地产租赁市场。打造更加优良的“双创”环境，加快推进省级创业孵化示范基地建设。全面加强社会保障体系建设，实施全民参保登记计划，继续推进被征地农民养老保险工程，加大社保扩面征缴力度。完善兜底性社会救助体系，推进社会救助提标提补，让困难群众生活得更加幸福、更有尊严。大力开展养老院服务质量建设专项行动和农村留守儿童关爱保护工作，确保老有所养、少有所育。着力推进城市交通治堵，加快城区道路网改造，促进小街小巷微循环，让百姓出行更便捷、更舒心。

积极发展社会事业。加快教育现代化建设，办好学前教育、职业教育和民办教育，统筹推进县域内义务教育基本均衡向义务教育优质均衡发展，科学调整教育网点布局，支持萍乡学院本科教学工作合格评估，积极申报萍乡技师学院，努力办好人民满意的教育。加快健康萍乡建设，全面推进萍乡卫生职业学院申办、中医药健康养生小镇建设、市三、四医院及安源区人民医院整合搬迁等重大项目，落实健康扶贫各项政策，加强基层医疗卫生体系和全科医生队伍建设，提升计划生育服务管理水平，倡导健康文明生活方式，实现萍乡医疗水平整体提高，人民健康水平不断提升。完善覆盖城乡的公共法律服务体系，提升法律服务水平。打造萍乡“文化名片”，做好红色文化、工业文化、禅宗文化、吴楚文化的挖掘、整理、保护和利用。创建全国公共文化服务体系示范区，深入实施文化惠民工程和乡镇、村公共文化设施提质达标工程，推进“四馆”建设，打造“一县一品”文化活动品牌。加强文艺精品创作，积极推动采茶戏《将军还乡》等作品申报国家“五个一工程奖”，推进萍乡文艺事业繁荣发展。积极推动全民健身，加快体育健身工程建设，推进社区、村级健身工程全覆盖。

全力抓好安全稳定工作。全面落实企业安全生产主体责任、地方和部门监管责任，推进“企业可防、政府可控”工程。突出抓好科技兴安，实现智慧安监全覆盖，建立安全生产与职业健康一体化监管执法，坚决杜绝重大以上安全生产事故，遏制较大事故，有效防范一般安全生产事故，提升安全生产总体水平。推进军民深度融合发展战略，扎实开展双拥共建活动。推进公安工作专业化、智能化，完善社会治安防控体系，依法打击和惩治黄赌毒黑拐骗等违法犯罪活动，进一步提高群众安全感和满意度。全力打造“阳光信访”“责任信访”“法治信访”，完善化解矛盾机制。着力抓好人民防空工作，推进人防地下指挥工程建设。加强食品药品安全监管，确保人民群众饮食用药安全。健全监测预防体系，提高灾害应对处置、救援救助能力。

（八）不断提高行政效能，建设法治廉洁政府

严格依法行政。全面贯彻依法治国战略，严格遵守宪法，尊崇法治，深入推进法治政府建设。完善行政决策机制，推进政府法律顾问制度，完善重大行政决策合法性审查机制，推进政府决策科学化、民主化、法制化。深化行政执法体制改革，加大“双随机一公开”工作力度。把法治理念贯穿政府工作全过程，提升政府工作人员法治思维和能力，使之成为法治政府建设的实践者、法治社会建设的引领者。

推进高效理政。以“最多跑一次”为目标，深化“放管服”改革，积极实施“三整合两缩减”，精简涉及生产和服务类行政许可事项和中介服务事项，确保年内完成市、县两级行政许可标准化建设。不断优化投资环境，为企业发展提供“店小二”式和“保姆”式服务。坚决落实收费目录清单管理制度，形成全社会监督的良好氛围。继续严格执行各项降费减负政策，切实为实体经济发展减负。

坚持清廉为政。扎实推进政府系统全面从严治党工作，严格执行党内问责和行政问责制度，推动全面从严治党向基层延伸。持续加强政府系统作风建设，持之以恒纠治“四风”问题。强化对公权力的制约和监督，确保权力规范有序运行。保持惩治腐败高压态势，建设风清气正的廉洁政府。

各位代表，时代赋予重托，奋进永不言止！让我们更加紧密地团结在以习近平同志为核心的党中央周围，在市委的坚强领导下，以党的十九大精神为指引，扎实工作，切实担负起新时代赋予的新使命，为全面建成小康社会，开创萍乡经济社会发展新局面而不懈奋斗!
